# Heart and Cardiovascular Involvement in Patients with Mucopolysaccharidosis Type IVA (Morquio-A Syndrome)

**DOI:** 10.1371/journal.pone.0162612

**Published:** 2016-09-09

**Authors:** Christoph Kampmann, Tariq Abu-Tair, Seyfullah Gökce, Christina Lampe, Jörg Reinke, Eugen Mengel, Julia B. Hennermann, Christiane M. Wiethoff

**Affiliations:** 1 Division of Pediatric Cardiology and Congenital Heart Diseases, Center for Diseases in Childhood and Adolescence, Mainz Medical University, Mainz, Germany; 2 Division of Metabolic Diseases (Villa Metabolica), Center for Diseases in Childhood and Adolescence, Mainz Medical University, Mainz, Germany; Cincinnati Children's Hospital Medical Center, UNITED STATES

## Abstract

**Background:**

Mucopolysaccharidosis (MPS) IVA is a rare lysosomal storage disorder with multiple skeletal and non-skeletal abnormalities requiring multiple surgical interventions. It is well known that patients with MPS IVA suffer from tachycardia, but cardiac and hemodynamic alterations have not been reported to date. We investigated the cardiovascular and hemodynamic alterations in patients with MPS IVA and developed a possible patho-mechanism for cardiovascular deterioration during anesthesia.

**Material and Methods:**

In this observational study, serial cardiac examinations were performed in 54 patients with MPS IVA who were followed at the Children’s Hospital of the Mainz Medical University (Mainz, Germany) between 1991 and 2014 (follow-up 1–24 years; median 5.8 years). Results were compared with data from a large central European cohort of more than 2000 healthy infants and children.

**Results:**

None of the patients had arterial hypertension, but 4% had evidence of increased pulmonary artery pressure. Patients developed aortic root extension up to 6.9 standard deviations above normal. Left-sided valve leaflet thickening occurred in 26 patients (five with valve disease). Patients had lower left ventricular dimensions (z: –1.02±0.1), lower stroke volumes (z: –2.3±0.17), lower left ventricular mass (z: –1.5±0.21), but higher wall thickness (z: +0.8±0.16), and higher work index (z: +2.5±0.2) compared to healthy control subjects. Cardiac output was preserved by an increase in heart rate of 21%. Sixty % of patients showed impaired diastolic filling; heart rate (99.0±1.8 vs. 92.0±2.1 bpm), age (18.0±1.8 vs. 14.2±1 years), and cardiothoracic ratio (61.6±3.6% vs. 55±4.2%) of these patients were higher compared to those with normal filling.

**Conclusions:**

The results of this study suggest an age-progressive disproportion of the intra-thoracic organs of patients with MPS IVA, which is accompanied by aortic root extension and thickened left ventricles, with reduced stroke volumes, impaired diastolic filling patterns, and increased heart rates.

## Introduction

Mucopolysaccharidoses (MPSs) are a group of rare inherited lysosomal storage disorders caused by deficiencies of functional enzymes that contribute to the degradation of glycosaminoglycans (GAGs). These enzyme deficiencies lead to progressive systemic deposition and storage of GAGs, finally resulting in multi-organ system dysfunction [1]. Depending on the particular GAGs that are deposited in tissues, a wide range of different organs can be affected across MPS types. Cardiac involvement has been reported among patients with MPS I, II, IVA, VI, and VII, with cardiac valve thickening, left ventricular hypertrophy, conduction abnormalities, coronary artery disease, and other vascular involvement occurring very early in life and, if unrecognized and untreated, contributing significantly to early mortality [2].

Among the different MPS diseases, MPS IVA (Morquio A syndrome; OMIM #253000) is a relatively rare condition, with an incidence of 0.15–0.47 per 10^5^ live births. MPS IVA is transmitted in an autosomal recessive manner. Deficiency in the enzyme N-acetylgalactosamine-6-sulfatase results in the lack of degradation and consequent storage of keratan sulfate and chondroitin 6-sulfate in various cell compartments (primarily lysosomes) of different organs (primarily cartilage and its extracellular matrix) and ultimately leads to incomplete enchondral ossification [3]. MPS IVA is clinically characterized by severe disproportionate dwarfism, dysostosis multiplex, anterior beaking of lumbar spine, pectus carinatum, flaring of the rib cage, dysplasia and laxity of joints, and corneal clouding among other signs, while intelligence remains normal. After 2–3 years of age, annual growth rate is dramatically slowed. Ultimate patient height is usually limited to around 100–130 cm and far below the 3rd percentile for growth. There is a degree of variability in disease severity, and patients with the severe phenotype do not usually survive past the second or third decade of life [4].

Cases of cardiac valve thickening, regurgitation, and/or stenosis have been reported in patients with MPS IVA [5–9]. In 1990, John and colleagues [6] reported echocardiographic data of a series of ten patients with MPS IVA. Six of these patients (60%) showed cardiac abnormalities, including mitral valve disease in five and aortic valve disease in four patients. Additionally, sinus tachycardia is a common feature in MPS IVA patients, which has gained substantial interest under treating physicians. However the mechanism underlying sinus tachycardia in these patients is unclear. Furthermore, patients with MPS IVA are facing a substantial number of surgical interventions, which may be complicated by an increased rate of cardio-pulmonary or cardiovascular morbidity, like in other MPS disorders like MPS I, MPS II and MPS VI [3]. To date, longitudinal changes in cardiac involvement and cardiac hemodynamics in patients with MPS IVA have not been reported in the literature and have not been investigated systematically in a larger cohort.

In light of the recent introduction of enzyme replacement therapies, it seems of particular interest to have a better understanding of the cardiac involvement and its hemodynamic consequences in patients with MPS IVA. It might also be helpful in managing the cardiovascular complications during anesthesia.

## Materials and Methods

### Patient selection

This study included cardiac data from all male and female patients with an enzymatically confirmed diagnosis of MPS IVA who were followed at the Children’s Hospital of the Mainz Medical University (Mainz, Germany) between 1991 and 2014. The study was performed in accordance with the ethical standards laid down in 1964 Declaration of Helsinki and its later amendments. The ethical review board of the university of Mainz waived the need for committee approval of the study as it concerned publication of results from normal exams of patients. All patients or their legal guardians provided written informed consent.

### Echocardiographic assessment

All echocardiographic examinations were performed according to the recommendations of the American Society of Echocardiography (ASE) [10]. Patients were placed in a left-sided recumbent position, and blood pressure measurements were performed on the right arm using a Dynamap monitor during echocardiographic examination. All analogue echocardiograms (15 echoes), stored on videotapes, were digitalized and reviewed by a single independent cardiologist. Left ventricular mass was calculated according to the Penn and ASE convention [11,12]. Left ventricular end-diastolic (LVEDV) and left ventricular end-systolic volumes (LVESV) were calculated according to Teichholz and colleagues [13], stroke volume (SV) was derived by subtracting LVESV from LVEDV. Cardiac output was calculated from SV times the simultaneously obtained heart rate, and cardiac Index was calculated from cardiac output per body surface area (BSA). Meridional peak-systolic wall stress (mPSS) was calculated as previously described [13–18], as was work index [19,20]. LV mass/LVEDV was calculated by LV mass per LVEDV [21], and LV mass was indexed to the BSA or per height power 2.7 [22,23].

The same calculations as described above were performed on source data from a large central European cohort of more than 2000 healthy infants and children [24]. From this cohort, regression analyses were calculated and analyzed in relation to the patient data. Deviations from normal were presented as z scores.

Diastolic function was classified as being normal, impaired, or restrictive depending on the E/A ratio and the deceleration time of the E-wave across the mitral valve [25]. Valve disease was defined according to Schmailzl [26] and Kampmann et al. [24]. Regarding stenosis, the peak gradient is given across the aortic valve while the mean gradient is given across the atrioventricular valves. Cardiothoracic ratio (CTR) was measured from posterior–anterior chest radiograms at inspiration. Increased pulmonary artery pressure was estimated by Doppler echocardiography either by physiological pulmonary regurgitation with a gradient > 20 mmHg in diastole or by physiological tricuspid regurgitation.

### Statistical methods

Data were recorded, and descriptive statistics were generated using SPSSv21 for Mac OS software. Groups were compared using the Student *t*-test or Fisher’s exact test, whichever was appropriate. A p value of <0.05 was considered to be statistically significant. Kaplan–Meier calculations and plots were generated in SPSS from the complete data set and split for patients presenting with and without valve thickening at first presentation to show the cumulative prevalence rate of valve disease dependent on valve thickening by age. Data are reported as mean ± standard deviation.

## Results

### Patient cohort

A total of 54 patients (32 males) with an enzymatically confirmed diagnosis of MPS IVA were examined. Apart from lower systolic and diastolic blood pressures in females, there were no significant differences in demographic or cardiac data between genders (Table 1). Patients’ origin was Turkish (n = 24), central European (n = 23), and Russian (n = 7). Longitudinal data comprised 225 examinations, with 36 patients having >4 examinations and 18 patients having >5 examinations. Median duration of follow-up was 5.8 years (range, 1–24 years).

**Table 1:** Baseline Biometric Characteristics of MPS IVA Patients (n = 54).

Characteristics	Male	Female	p Value
	n = 32	n = 22	
Age (yrs)	14.2±11.1 (10.1 to 18.2)	15.8±12.7 (10.2 to 21.5)	NS
Height (cm)	112±21.3 (104 to 119)	110±21.4 (100 to120)	NS
Weight (kg)	27.9±16 (21 to 33.6)	26.5±13.6 (19.9 to 29.6)	NS
Body surface area (m^2^)	1.12±0.4 (0.95 to 1.30)	1.08±0.34 (0.91 to 1.24)	NS
Body mass index (kg/m^2^)	21.1±5.2 (19.23 to 22.9)	20.7±6.1 (18 to 23.4)	NS
Systolic blood pressure (mmHg)	113±16 (107 to 119)	104±10.5 (99 to108)	0.017
Diastolic blood pressure (mmHg)	75±12.4 (71 to 80)	67±9 (64 to 71)	0.01
Mean arterial pressure (mmHg)	89±11 (84.4 to 93)	93±17.4 (77 to 88)	0.05
Hart rate (bpm)	96±19.5 (88 to 104)	93±18 (83 to 101)	NS
Cardiothoracic ratio (%)	58.3±5.6 (55 to 62)^a^	59.6±5.3 (57 to 62)^b^	NS

Values are mean ± standard deviation (95% confidence interval).

^a^n = 24

^b^n = 21

### Involvement of the systemic arterial system

Out of 225 measurements of systolic and diastolic blood pressure, seven were indicating arterial hypertension (systolic highest 148 mmHg, diastolic highest 100 mmHg), but none of these measurements were reproducible.

**Fig 1:**
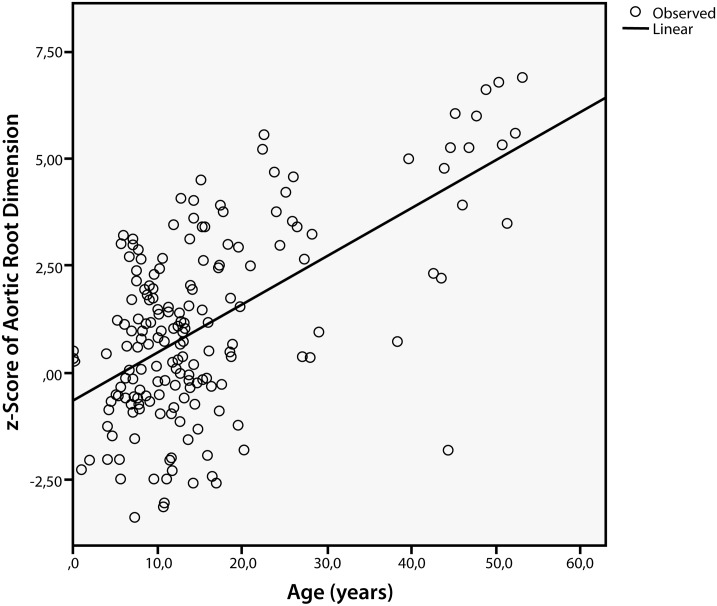
Correlation Between Age and z Score of Aortic Root Dimensions in MPS IVA Patients. Patients with MPS IVA develop a dilatation of the aortic root in relation to BSA with ageing (r2 = 0.586; ANOVA F:95.717; p<0.005). Created using SPSS software.

At first examination female patients had slightly smaller aortic root dimensions than males (aortic diameter z score: females: 0.3±2.8 (95% confidence interval [CI]: -0.7–3.4) vs. males: 1.1±2 (95% CI: -0.3–3.1)). After finishing growth, the z scores differed significantly between younger and older patients (aortic diameter z score <18 years of age: 0.48±1.7 and ≥ 18 years of age: 3.17±2.1; p<0.0001), and there was a strong correlation between age and z score of aortic root dimensions normalized to BSA (r^2^ = 0.586, ANOVA: p<0.001) (Fig 1), a weaker correlation between blood pressure (mean arterial pressure: r^2^ = 0.34, ANOVA: p<0.001), but no correlation to the CTR.

### Involvement of the pulmonary arterial system

Two out of 54 patients showed a reproducible increase in pulmonary artery pressure. This was not related to valve disease or valve thickening of the mitral valve. However, patients with increased pulmonary artery pressure had higher z scores for heart rate and CTR than those without signs of increased pulmonary artery pressure (HR z score: 2.2±0.6 vs. 1.2±0.9; p = 0.019 and CTR: 64.6±6 vs. 57.8±4; p = 0.038).

### Valvular involvement

**Fig 2:**
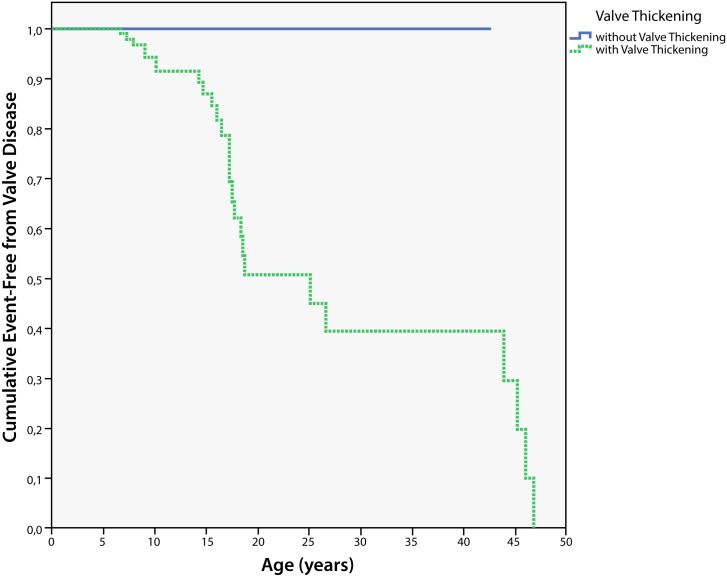
Cumulative Prevalence of Valve Disease in MPS IVA Patients. Patients presenting with (dotted line) or without (solid line) valve thickening at first examination. Bars on the line indicate patients without valve thickening at first examination. Early valve thickening predisposes to the development of valve disease at later age. Created using SPSS software.

None of the patients had detectable thickening of cardiac valve leaflets or other than normal “physiological” regurgitation or stenosis of right-sided heart valves (tricuspid or pulmonary valves) either at baseline or during follow-up. At first examination, 25 patients (mean age 15±10.3 years) had normal shaped left-sided valves (mitral and aortic valves) while 26 patients (mean age 11.6±9 years) showed minimal to mild thickened mitral and/or aortic valve leaflets. Five patients had overt left-sided valve disease, including a 47-year-old patient with a myxomatous calcified bicuspid aortic valve with moderate stenosis and regurgitation (Table 2). The five patients with overt valve disease were older (30.0±19.9 vs. 12.5±8.8 years, p = 0.002) and had a higher body mass index (22.3±5.6 vs. 26.3±9.96 kg/m^2^; p = 0.002) than those without valve disease, but were of comparable height (108.06±15.9 vs. 110.4±13.5 cm; p = 0.45). Patients without left-sided valvular thickening at a mean age of 15 years did not develop valvular disease during the median follow-up of 5.8 years, while those patients who developed overt valvular disease already had valvular thickening at first presentation (p<0.0001; log-rank Mantel–Cox) (Fig 2).

**Table 2:** Valve Involvement in MPS IVA Patients (n = 54).

Valve	Regurgitation	Stenosis
Tricuspid	0	0
Pulmonary	0	0
Mitral	2 (≥1°)	0 (≥5 mmHg mean)
Aortic	3 (≥2°)	2 (≤15 mmHg mean)
	1 (3°)	1 (35 mmHg mean)

Values are number of patients.

### Involvement of the cardiovascular system

At first examination, all but three patients (51/54; 94.4%) had accelerated heart rates, with a mean of 94.5±2.7 bpm (range, 60–135 bpm) at a mean age of 11.4±1.6 years, which was 21% above normal. Heart rate was more closely inversely correlated with patient height (r^2^ = –0.44) than with age (r^2^ = –0.34). During follow-up, heart rate did not decrease and remained unchanged: 94.6±2.9 bpm at first examination and 99.4±3.6 bpm at last examination (ANOVA; p = 0.89).

**Fig 3:**
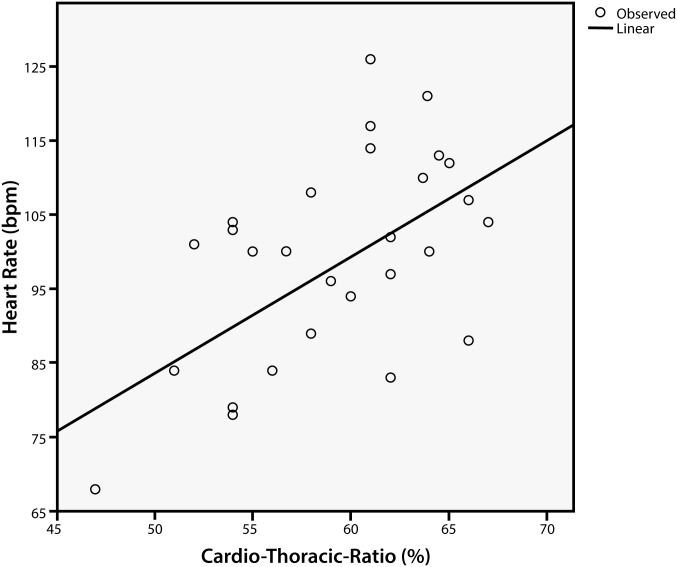
Correlation Between Heart Rate and Cardiothoracic Ratio (CTR) in MPS IVA Patients. Patients with higher CTR had increased heart rates (r^2^ = 0.52; CTR = 0.171*HR + 42.43; ANOVA F = 9.9, p = 0.04). Created using SPSS software.

A total of 45 chest radiographs for 36 patients were available during follow-up. Heart size in relation to the inner thoracic diameter was on average 59.8±5.41% (range, 47–67%), indicating an enlarged heart in relation to the thorax diameter. Only one patient showed a CTR<50% (normal values range from 40% to 50%; (28)): this patient had a height of 1.83 m, which indicates an attenuated, very mild form of MPS IVA. No radiographs were available in patients <5 years of age. CTR was inversely correlated with patient height (p = 0.04). However, heart rate showed a linear correlation with CTR (r^2^ = 0.52, p = 0.04) (Fig 3).

Although CTR was increased, patients had a smaller BSA-indexed left ventricular end-diastolic dimension (LVIDi z score –1.02±0.1; 95% CI, –1.2 to –0.8), LVEDV (LVEDVi z score –0.64±0.14; 95% CI: –0.9 to –0.34), SV (SVi z score: –2.3±0.17; 95% CI: –2.63 to –1.98), and left ventricular mass (LVMi z score –1.5±0.21; 95% CI: –1.86 to –1.0) than healthy controls. However, cardiac index (z score –0.5±0.08; 95% CI: –0.6 to –0.3) and mPSS (44.95±1.3 g/cm^2^; 95% CI: 38.8 to 50) were preserved while work index (z score 2.5±0.2; 95% CI: 2.09 to 2.9) was substantially higher compared to healthy controls.

The left ventricles were normally shaped (relative wall thickness <0.45 and LVMi <110 g/m^2^) in 32 patients. 19 patients showed concentric remodeling (relative wall thickness ≥0.45 and LVMI <110 g/m^2^), and three patients exhibited valvular disease with mild left ventricular hypertrophy (LVMi ≥110 g/m^2^).

All patients had normal systolic function parameters: ejection fraction (71.6±0.6%; 95% CI: 70.4 to 72.9%) and fractional shortening (40.6±0.5%; 95% CI: 39.6 to 41.6%). Information on diastolic filling patterns was available for 28 patients: 12 had normal diastolic filling patterns while 16 patients showed moderate impaired, non-restrictive filling patterns. Diastolic impairment was more common in patients with concentric remodeling (9 of 10 patients at first examination) than in patients with normally shaped left ventricles (5 of 14 patients at first exemption) (chi-square 7.95; p = 0.047). Patients with impaired filling had higher heart rates (99.0±1.8 vs. 92±2.1 bpm, p = 0.009) and were older (18.02±1.8 vs. 14.24±1.0 years; p = 0.031) than those without impaired filling. CTR was higher in patients with diastolic impairment than in those with normal filling patterns (n = 17; 61.6±3.6 vs. 55±4.2, p = 0.009).

When patients were grouped according to absence or presence of valve thickening, myocardial changes were more prominent in those without valve thickening regarding the z scores for LVIDi, LVEDVi, SVi, and LVMi. Patients with valve thickening had higher relative wall thickness, mean wall thickness, and LVMi but comparable functional parameters, thereby requiring higher LVM per EDV and higher heart rate-normalized work index to keep mPSS and cardiac output constant (Table 3).

**Table 3:** Cardiac Data for MPS IVA Patients with and without Valvular Thickening.

Cardiac Parameter	No Valve Thickening	Valve Thickening	p Value
	n = 26	n = 28	
Heart rate (bpm)	96.5±17.4 (89 to 103.6)	94.5±18.2 (87 to 102)	NS
Heart rate (z score)	1.38±1.4 (0.8 to 2.0)	1.2±1.5 (0.6 to 1.8)	NS
LVIDi (z score)	–1.17±1.3 (–1.4 to –0.98)	-0.37±1.6 (–1.0 to +0.25)	0.021
LVEDVi (z score)	-0.76±1.7 (–1.0 to –0.47)	0.05±2.1 (–0.73 to +0.83)	0.05
SVi (z score)	-2.38±2.15 (–2.74 to –2.0)	–2.0±2.5 (–3.0 to –1.1)	NS
Cardiac index (z score)	–0.56±1 (–0.74 to –0.38)	-0.2±1.29 (–0.7 to +0.25)	NS
LVMi (z score)	-1.68±2.8 (–2.16 to –1.2)	–0.3±2.3 (–1.1 to +0.6)	0.008
Work index (z score)	2.35±2.5 (1.9 to 2.76)	3.2±3.18 (2.02 to 4.38)	0.1
Heart rate-normalized work index(kg•m/100 bpm)	172.2±90 (157 to 187.4)	223.6±125 (176.7 to 270.5)	0.04
Relative wall thickness (cm)	0.4±0.09 (0.388 to 0.42)	0.45±0.1 (0.4 to 0.48)	0.038
Mean wall thickness (cm)	0.71±0.15 (0.68 to 0.73)	0.87±0.18 (0.8 to 0.94)	0.0001
LVM per EDV (g/ml)	1.27±0.47 (1.2 to 1.36)	1.56±0.48 (1.37 to 1.74)	0.007
mPSS (g/cm^2^)	45±17.5 (42.1 to 48)	44.5±15.1 (38.8 to 50)	NS
Cardiothoracic ratio (%)	60.4±4.8 (58 to 62.7)^a^	59.2±5.3 (54.3 to 64.2)^b^	NS
Aortic diameter (cm)	2.17±0.46 (2.05 to 2.28)	2.24±0.43 (2.16 to 2.32)	NS
Aortic diameter (z score)	0.67±2.1 (0.13 to 1.2)	1.31±2.2 (0.91 to 1.7)	0.054

Values are mean ± standard deviation (95% confidence interval).

^a^n = 29

^b^n = 15

BSA = body surface area; EDV = end-diastolic volume; LVEDVi = BSA-indexed left ventricular end-diastolic volume; LVIDi = BSA-indexed left ventricular internal dimension at end-diastole; LVMi = BSA-indexed left ventricular mass; mPSS = meridional peak-systolic wall stress; SVi = BSA-indexed stroke volume.

### Comorbidities and treatment

The authors could not find any co-morbidities that could explain the reported alterations other than Morquio A disease. Only in one patient a myxematous bicuspid aortic valve was present (see Discussion). None of the patients had a family history of aortic wall diseases. None of the patients was treated for aortic ectasia or hypertension. Three patients were treated for severe sinus tachycardia with low dose beta blockers 0.02–0.05 mg/kg body weight bisopolol. It is unclear if this might have affected the observed aortic wall changes. All baseline examinations were performed in patients who were naïve to ERT with elosulfase alfa. Fourteen patients with a median age of 10.1 years (mean: 11.1 years) (25.9%) started ERT during follow-up. Mean ERT follow-up time was 2.6 years (median 2.6 years, longest ERT time 3.4 years) and these patients contributed 49 examinations (21.8%).

## Discussion

This is the first report of cardiovascular involvement in Morquio A syndrome. Compared to other MPS types, the majority of patients with MPS IVA face a severe dysmorphic stature with predominantly skeletal problems [3]. Because of the severity of the skeletal problems, non-skeletal features such as respiratory and cardiovascular impairment have received little attention [3]. Arterial hypertension could not be demonstrated in the presented cohort of patients, although there are some alterations in the systemic arterial vasculature, as evident from a progression of aortic root growth in relation to age. Increased pulmonary artery pressure was estimated to be present in approximately 4% of patients, clearly related to the severe dysmorphic thorax and not to valvular disease, although mitral valve disease may aggravate the development of pulmonary hypertension.

The main GAG storage products in MPS I, II, and VI are dermatan, heparan, and chondroitin sulfates, respectively, while for MPS IVA, the main storage products are keratan sulfate and variable quantities of chondroitin sulfate [3]. Cardiac valve alterations may therefore be less prominent in MPS IVA than in MPS I, II, and VI.

Right-sided valvular changes, such as leaflet thickening resulting in regurgitation or stenosis, are uncommon and could not be demonstrated in the present cohort of patients with MPS IVA. However, approximately half of the patients demonstrated a small increase in the thickness of left-sided valve leaflets, which was readily detected by echocardiography at around 10 years of age. None of the patients with normally shaped left-sided valves developed left-sided valve disease in terms of relevant regurgitation or stenosis during follow-up. However, patients who already had valvular thickening at a young age (11.6±9 years) tended to develop valve disease during follow-up. The aortic valve was most prominently affected, leading to regurgitation rather than stenosis. One patient exhibited moderate aortic stenosis with myxomatous calcification of the aorta as well as a bicuspid aortic valve; this patient may have had two different conditions leading to valve disease, with MPS IVA aggravating the problems caused by the bicuspid aortic valve.

All but one patient showed an increased heart size relative to inner thoracic diameter, indicating “cardiomegaly”. However, after BSA adjustment, patients with MPS IVA had smaller end-diastolic dimensions of the heart, resulting in lower stroke volume compared to healthy subjects. This suggests that the physiological growth of the heart (and its adaptation) may be limited by the small intra-thoracic space and that heart growth may finally aggravate the thoracic deformities by developing a voissure and pectus carinatum. It is known that patients with “small heart syndrome” suffer more from chronic fatigue than control patients with a normal heart size [27].

Stroke volume indexed to BSA deviated from that of healthy controls by –18.8 ml (z score –2.4; i.e., 29.3% lower). However, cardiac index was normal compared to healthy subjects. To overcome and cope with the reduced stroke volume, the heart can either increase systolic function or accelerate heart rate. As systolic function parameters were normal, cardiac index seems to be maintained in MPS IVA patients by increasing heart rates at rest. This adjustment is also demonstrated by the linear correlation between heart rate and CTR. Patients in whom diastolic filling is additionally impaired by an increased CTR show an increased heart rate independent of age. This increase leads to a higher work load, needing more muscle mass per ml of volume to keep the wall stress of the myocardium constant, resulting in higher energy consumption of the myocardium.

An autopsy case of a patient with MPS IVA has been published recently [28]. The authors reported a heart weight of 200 g in a 20-year-old male patient (height 91.4 cm and body weight 20.3 kg). With a heart weight to body length ratio of 2.19, the heart was judged as being normal (1.84±0.31). However, in relation to body weight, the heart’s weight of 200 g seemed to be above the upper limit of normal (maximum critical heart weight: 7.5 g/kg body weight; here 152 g). Apart from valvular and aortic involvement, no other cardiac changes were reported, although the authors described a very high density of relatively small myocardial cells. This description supports our findings of a thick-walled and small-lumen left ventricle with reduced diastolic compliance, which can be complicated by a thickened endocardiac membrane.

The role and influence of ERT with elosulfase alfa on the presented cardiovascular findings may be neglectable. In the very short period during which 14 patients received ERT (median follow-up 2.6 years), there were no significant changes notable in heart rate, wall thickness, diastolic filling properties, shape of the valves, or extension of the aortic root.

Our findings may have substantial implications for the understanding of the cardiovascular situation among patients with MPS IVA. Sinus “tachycardia” in MPS IVA patients seems to be a physiological reflex to maintain cardiac output in a small-sized heart with impaired filling patterns rather than an “arrhythmia” or a result of a sinus node or autonomic dysfunction. Pharmaceutical interventions to reduce heart rate should be considered with caution in these patients because of the possible reduction in cardiac output.

During their lifespan, MPS IVA patients need a substantial number of surgical procedures requiring general anesthesia. Many anesthetic drugs cause a decrease in vascular resistance to which, MPS IVA patients will react with a more than proportional increase in heart rate and an accompanying continuous deterioration in diastolic filling properties. Furthermore, the hemodynamic alterations in MPS IVA patients may aggrevate the well-recognized reduced exercise capacity of these patients as a higher work load of the heart and a higher heart rate at rest result in a reduced heart rate spectrum and therefore a reduced ability to modulate cardiac output.

## Conclusions

The results of the present study suggest that the progressively worsening disproportion of intra-thoracic organs with ageing in patients with MPS IVA may be due to kypho-scoliotic thorax deformity and pectus carinatum. This may result in inadequate growth of the aorta and impaired adaptation of the left ventricle with a relatively thickened, but small left ventricle leading to a) a reduced stroke volume, which is compensated by an increased heart rate and an increased work load to maintain an appropriate cardiac output and b) an impaired diastolic filling pattern due to the relatively thick-walled ventricle and thickened endocardial membrane. Impaired lung function, increases in pulmonary artery pressure and a limited ability to modulate heart rate and cardiac output may (partly) explain the progressive reduction in exercise capacity seen in patients with MPS IVA. More research is warranted to confirm the findings of this study and the association with exercise capacity.
